# Limited Diagnostic Utility of Postoperative C-Reactive Protein for Early Detection of Surgical Site Infections Following Posterior Scoliosis Correction Surgery

**DOI:** 10.3390/jcm14217511

**Published:** 2025-10-23

**Authors:** Mateusz Zebrowski, Maria Czubak-Wrzosek, Jaroslaw Czubak, Marcin Tyrakowski

**Affiliations:** 1 Department of Spine Disorders and Orthopedics, Centre of Postgraduate Medical Education, Gruca Orthopaedic and Trauma Teaching Hospital, 05-400 Otwock, Poland; czubakwrzosek@gmail.com; 2 Department of Orthopedics, Pediatric Orthopedics and Traumatology, Centre of Postgraduate Medical Education, Gruca Orthopaedic and Trauma Teaching Hospital, 05-400 Otwock, Poland; kootd@cmkp.edu.pl

**Keywords:** surgical site infection, C-reactive protein, scoliosis, posterior spinal fusion, spine surgery

## Abstract

**Background/Objectives:** Surgical site infection (SSI) is a serious complication following scoliosis correction surgery. While CRP monitoring is commonly used after major orthopedic procedures such as joint arthroplasty, its utility for early SSI detection after scoliosis surgery remains unclear. The aim of this study was to evaluate the diagnostic value of postoperative serum C-reactive protein (CRP) concentrations for identifying early surgical site infections (SSI) in patients undergoing scoliosis correction surgery. **Methods:** A retrospective analysis was performed on a prospectively collected database of 358 patients who underwent posterior spinal fusion for scoliosis between 2014 and 2024 at a single orthopedic center. Patients were divided into idiopathic scoliosis (IS, *n* = 268) and non-idiopathic scoliosis (N-IS, *n* = 90) groups. Serum CRP concentrations were measured postoperatively on days 2 and 4 or 3 and 5. The incidence of early SSI and postoperative CRP trends were compared between patients with and without SSI. **Results:** The overall early SSI rate was 3.91% (IS: 2.24%, N-IS: 8.89%). A clear postoperative decline in CRP was observed in the non-SSI group. In contrast, only 2 patients with confirmed SSI demonstrated a rising CRP trend in the early postoperative period, indicating poor sensitivity of this marker for early infection detection. **Conclusions:** Postoperative CRP dynamics showed limited diagnostic value for early SSI detection. A rising CRP occurred in only a minority of infected cases. Therefore, CRP should not be used as a standalone marker, and clinical assessment remains essential for early SSI diagnosis.

## 1. Introduction

Surgical Site Infection (SSI) is one of the most serious complications related to the surgical correction of pediatric scoliosis. Its occurrence may result in prolonged hospitalization, the use of long-term antibiotic treatment, repeated irrigation, and debridement surgeries. Persistent infection can lead to the necessity of implant removal in the most severe cases of SSI. This may result in a loss of spinal correction and decreased patient satisfaction [1,2,3,4].

The Centers for Disease Control and Prevention (CDC) classifies SSIs as either superficial or deep. Superficial SSIs affect the skin and subcutaneous tissue at the incision site and typically manifest within 30 days postoperatively. Deep SSIs, on the other hand, affect tissues beneath the paraspinal fascia and develop within 90 days, or, according to some authors, even up to one year after surgery [5].

Reported incidence rates of SSIs following scoliosis correction in patients with adolescent idiopathic scoliosis range from 0.5% to 6.7% [2]. In patients with neuromuscular scoliosis, the incidence is notably higher, ranging from 4.3% to 14.3% [2].

The diagnosis of SSI in pediatric patients after scoliosis correction is primarily based on clinical presentation [2]. However, there is a lack of literature evaluating the utility of laboratory markers as tools for early detection of infection in this specific patient population [6].

C-Reactive Protein (CRP) is an acute-phase protein synthesized by hepatocytes and is widely used in orthopedics for the early identification of infectious complications due to its predictable kinetics and rapid elevation in response to inflammation. Monitoring CRP levels in the early postoperative period is a common practice in adult spinal surgery [7,8] as well as in joint arthroplasty [9,10].

CRP typically peaks on the 2nd–3rd postoperative day and subsequently declines in patients with an uncomplicated course [7]. A secondary elevation or failure of CRP to decrease has been suggested as an early indicator of infection in adult spinal surgery and arthroplasty [8,11]. However, evidence regarding the diagnostic value of CRP for early detection of SSI after pediatric scoliosis correction remains scarce. Therefore, the aim of this study was to evaluate the diagnostic value of postoperative serum C-reactive protein (CRP) levels for the early identification of surgical site infections in patients undergoing surgery for idiopathic and non-idiopathic scoliosis.

## 2. Materials and Methods

A retrospective analysis was conducted using a prospectively collected database of patients who underwent scoliosis surgery at a single orthopedic center. The study included 358 patients who underwent posterior spinal instrumented fusion between October 2014 and July 2024. Demographic data—including age, height, weight, BMI, and sex—as well as clinical data, such as scoliosis type (idiopathic vs. non-idiopathic), the presence of surgical site infection (SSI), and serum C-reactive protein (CRP) concentrations, were recorded.

The study was conducted in accordance with the Declaration of Helsinki and approved by the Bioethics Committee at the Centre of Postgraduate Medical Education in Warsaw, Poland (Resolution No. 66/2023, dated 8 March 2023). An amendment extending the study period through July 2024 and increasing the sample size was approved by the same committee (Resolution No. 87/2025, dated 10 September 2025). Owing to the retrospective design and use of de-identified data, the requirement for individual informed consent was waived.

We included patients with idiopathic or non-idiopathic scoliosis undergoing posterior spinal fusion via a posterior approach. Both pediatric and adult patients (≥18 years) were eligible for inclusion. There were no age-related exclusion criteria; all patients who underwent scoliosis correction surgery during the study period were included. Patients with degenerative scoliosis and those undergoing posterior spinal fusion for other indications (e.g., fractures, spinal infection) were excluded.

Patients were categorized into two primary groups based on the etiology of scoliosis: idiopathic scoliosis (IS, *n* = 268) or non-idiopathic scoliosis (N-IS, *n* = 90). The N-IS group included 48 patients with neuromuscular scoliosis (comprising 41 with cerebral palsy, 5 with myelomeningocele, and 2 with other neuromuscular disorders), 35 with syndromic scoliosis, 4 with secondary scoliosis, and 3 with congenital scoliosis. Within each group, patients who developed SSI were identified.

From 2014 to 2018, serum CRP concentrations were measured on postoperative days 3 and 5. Beginning in 2019, the measurement protocol was modified to postoperative days 2 and 4. This approach aligned with the hospital’s internal protocol for early postoperative infection surveillance following major joint arthroplasty.

Differences in CRP values and their perioperative dynamics were compared between patients who developed early SSI and those who did not. Early SSI was defined as an infection occurring within 30 days postoperatively. The diagnosis was based primarily on clinical symptoms, including delayed wound healing, erythema around the incision, wound discharge, fever of unknown origin, persistent postoperative pain, or a sudden onset of pain during the postoperative period. Laboratory findings, particularly serum CRP concentrations and their postoperative trends, were also assessed to support the diagnosis.

Statistical analyses were performed using Microsoft Excel for Microsoft 365 MSO, Version 2504 (Microsoft Corp., Redmond, WA, USA) and Statistica 14.1 (TIBCO Software Inc., San Ramon, CA, USA). The normality of the distribution of quantitative variables was assessed using the Shapiro–Wilk test. Due to the small number of patients with SSI, non-parametric tests—including the Wilcoxon signed-rank test and the Mann–Whitney *U* test—were used to evaluate the statistical significance of differences between groups.

Cut-off values for CRP on postoperative days (POD) 2/3 and POD 4/5 were determined using receiver operating characteristic (ROC) curve analysis and Youden’s index. Sensitivity, specificity, positive predictive value (PPV), and negative predictive value (NPV) were calculated for the identified cut-off values. A *p*-value of <0.05 was considered statistically significant.

We also performed a post hoc power analysis and a prospective sample-size estimation for comparing two independent proportions using Statistica 14.1 (TIBCO Software Inc.; Two Proportions, *Z*-Test). Calculations assumed a two-tailed α = 0.05, unequal allocation fixed to the observed ratio of controls: cases (~24:1), and no continuity correction.

## 3. Results

A total of 358 patients were retrospectively included in the study, comprising 268 with idiopathic scoliosis and 90 with non-idiopathic scoliosis. Serum CRP concentrations were analyzed on postoperative days 2 and 4 or days 3 and 5. The study assessed differences in CRP values and their dynamics between patients who developed early surgical site infections (SSI) and those without this complication.

In the idiopathic scoliosis subgroup (AIS, *n* = 270), the mean age was 15.78 years (range 7.59–36.99); 36 patients (13.3%) were adults (adult subgroup: mean 23.44 years, range 18.13–35.99). In the non-idiopathic subgroup (N-IS, *n* = 90), the mean age was 15.01 years (range 7.18–46.88); 9 patients (10.0%) were adults (adult subgroup: mean 24.29 years, range 18.59–48.83). The difference in mean age between AIS and N-IS patients was not statistically significant (*p* = 0.131). Overall, adults represented 45/360 (12.5%) of the study population.

The mean BMI in the N-IS group was 17.72 kg/m^2^ (range: 9.28–37.28), whereas in the IS group, it was 19.81 kg/m^2^ (range: 11.92–35.63). This difference was statistically significant (*p* < 0.001). In the IS group, there were 65 males and 203 females, while in the N-IS group, there were 28 males and 62 females. The difference in sex distribution between the groups was not statistically significant (*p* = 0.252). The mean body weight in the IS group was 53.73 kg (range: 19–115), compared to 41.5 kg (range: 13.2–92) in the N-IS group. This difference approached but did not reach statistical significance (*p* = 0.077) (Table 1).

**Figure 1:**
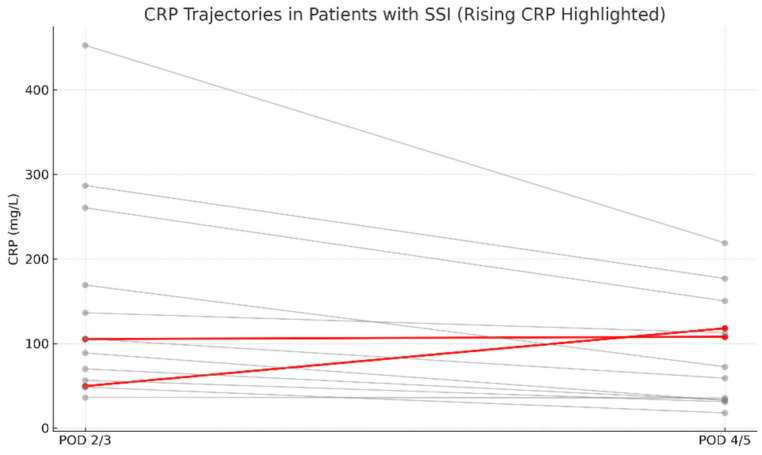
Individual serum CRP trajectories in patients with early surgical site infection (SSI) between postoperative days (POD) 2/3 and 4/5. Red lines indicate patients with rising CRP levels, suggestive of ongoing or worsening infection. Grey lines represent patients showing a typical postoperative CRP decline.

**Figure 2:**
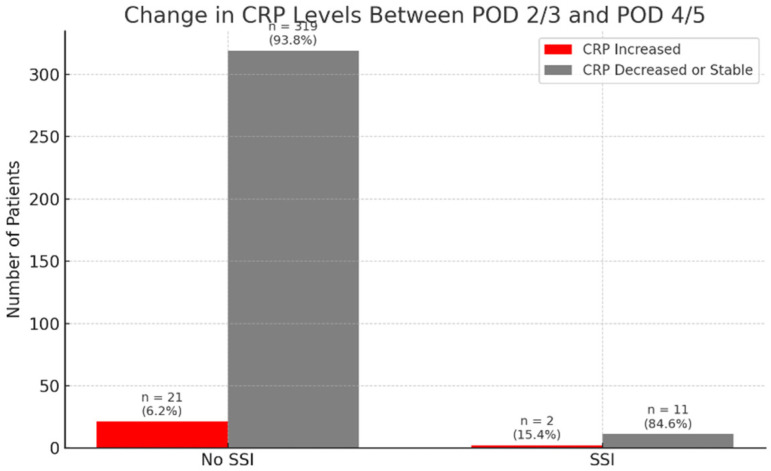
Distribution of patients with and without early SSI according to changes in serum CRP concentrations between postoperative days (POD) 2/3 and 4/5. Red bars indicate patients with an increase in CRP levels; grey bars indicate stable or decreasing values.

In the IS group, SSI occurred in 6 cases (6/268; 2.24%), whereas in the N-IS group, 8 cases were identified (8/90; 8.89%). The overall incidence of early SSI in the study cohort was 3.91%. All surgical site infections were diagnosed within 30 days postoperatively and thus met the CDC criteria for early SSI. Out of 358 patients, 14 developed SSI, while 344 did not. Complete CRP data for the relevant postoperative days were available for 13 patients in the SSI group and 342 in the non-SSI group. Among patients with SSI, only 2 out of 13 (15.4%) showed an absolute increase in CRP levels between postoperative days (POD) 2/3 and POD 4/5 (Figure 1). In comparison, a rise in serum CRP concentrations was observed in 21 out of 342 non-SSI patients (6.2%) (Figure 2). These findings indicate that an isolated increase in CRP during the early postoperative period has low sensitivity and limited diagnostic value as a standalone predictor of early SSI.

Based on the observed proportions of CRP increase between postoperative days 2/3 and 4/5 (SSI: 14.3% [2/14] vs. non-SSI: 6.2% [21/340]), the achieved power was ~31%, indicating limited sensitivity to detect small-to-moderate effects. Under the same assumptions, ~96 SSI cases and ~2342 controls would be required to achieve 80% power.

Among the 14 identified cases of SSI, 2 were managed conservatively, while the remaining 12 required surgical intervention. In all patients, intraoperative specimens were collected for microbiological analysis. Postoperatively, empirical antibiotic therapy was administered to all patients. In 50% of cases, cultures were positive, and antibiotic therapy was subsequently tailored according to the antibiogram. The isolated pathogens included *Staphylococcus epidermidis* (3 cases), *Propionibacterium* (*Cutibacterium*) *acnes*, *Enterobacter cloacae*, and *Anaerococcus tetradius*.

The mean (range) length of hospital stay [days] was: IS, 8.1 (5–32); N-IS, 10.8 (5–30); SSI, 18.1 (6–32); non-SSI, 8.3 (5–32). Length of stay was markedly longer in patients with SSI; some cases presented within the first postoperative days, prolonging the index stay, whereas others required a later readmission.

**Figure 3:**
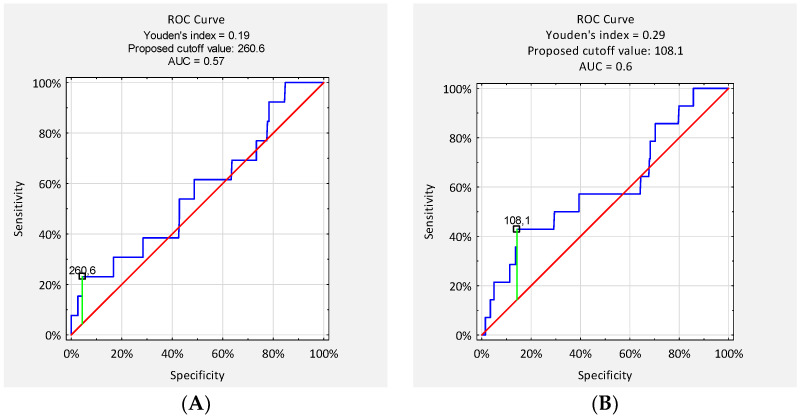
Receiver operating characteristic (ROC) curves for serum CRP concentrations on (**A**) postoperative days (POD) 2/3 and (**B**) POD 4/5 in the entire study cohort. AUC: area under the curve. Colors: blue—ROC curve; red—no-discrimination reference line; green—proposed cut-off value (Youden index).

Receiver operating characteristic (ROC) analysis was conducted to assess the diagnostic performance of serum CRP concentrations on postoperative days (POD) 2/3 and 4/5 in detecting early surgical site infection (SSI). The area under the curve (AUC) for CRP on POD 2/3 was 0.57, and on POD 4/5 it was 0.60, indicating poor to moderate discriminatory ability. The optimal CRP cut-off value on POD 2/3, determined using the Youden index, was 260.6 mg/L, resulting in a sensitivity of 23% and specificity of 96%. The corresponding positive predictive value (PPV) was 17%, and the negative predictive value (NPV) was 97%. On POD 4/5, the optimal cut-off was 108.1 mg/L, with a sensitivity of 46% and specificity of 86%. The PPV for this threshold was 11%, while the NPV reached 98% (Figure 3).

Group measured on POD 2 and POD 4 (Table 2).

Among patients with IS without SSI, mean CRP was 78.93 mg/L ± 52.40 (range: 5.9–320.5) on POD 2 and 51.72 mg/L ± 42.41 (range: 2.6–258.8) on POD 4; the decrease was significant (*p* < 0.05; *p* = 1.173 × 10^−8^). In patients with IS with early SSI, mean CRP was 61.96 mg/L ± 27.10 (range: 36.3–105.3) on POD 2 and 55.93 mg/L ± 44.88 (range: 18.1–118.1) on POD 4; the difference was not significant (*p* = 0.89). In the N-IS cohort without SSI, mean CRP was 139.28 mg/L ± 79.26 (range: 3.8–370.4) on POD 2 and 85.15 mg/L ± 60.08 (range: 2.4–246.2) on POD 4; the decrease was significant (*p* < 0.01). In N-IS with SSI, mean CRP was 191.88 mg/L ± 180.20 (range: 56.7–452.7) on POD 2 and 88.90 mg/L ± 88.82 (range: 32.2–219.0) on POD 4; the decrease did not reach statistical significance (*p* = 0.069).

Group measured on POD 3 and POD 5 (Table 3).

Among patients with IS without SSI, mean CRP was 148.44 mg/L ± 73.52 (range: 26.2–355.4) on POD 3 and 59.97 mg/L ± 40.76 (range: 1.2–177.9) on POD 5; the decrease was significant (*p* < 0.05; *p* = 1.003 × 10^−11^). No IS patients with SSI were included in this group. In the N-IS cohort without SSI, mean CRP was 177.68 mg/L ± 79.88 (range: 48.7–368.2) on POD 3 and 81.58 mg/L ± 44.61 (range: 11.5–194.8) on POD 5; the decrease was significant (*p* < 0.01). In N-IS with SSI, mean CRP was 197.40 mg/L ± 89.69 (range: 105.7–286.9) on POD 3 and 124.83 mg/L ± 51.12 (range: 59.1–177.0) on POD 5; the decrease did not reach statistical significance (*p* = 0.069).

When comparing homogeneous patient groups diagnosed with IS, two subgroups were identified: patients without SSI (262/268; 97.76%) and those with confirmed SSI (6/268; 2.24%). To evaluate the potential diagnostic value of serum CRP concentrations in detecting early SSI, CRP levels were compared between the groups within the measurement set in which CRP was obtained on POD 2 and POD 4. The Mann–Whitney U test was used to assess statistical significance.

On POD 2, the comparison yielded a *Z*-score of 0.601 and a *p*-value of 0.556. On POD 4, the *Z*-score was −0.094 with a *p*-value of 0.924. These results indicate no statistically significant differences in CRP levels between patients with and without early SSI on either POD 2 or POD 4 (Table 4).

In the separate IS group in which CRP was measured on POD 3 and POD 5, no cases of SSI occurred; therefore, no between-group comparison was possible and this group is not represented in Table 4.

Among patients with non-idiopathic scoliosis (N-IS), early surgical site infection (SSI) occurred in 8 out of 90 cases (8.89%). To assess the diagnostic utility of serum CRP concentrations, CRP levels on specific postoperative days were compared between patients with and without SSI. In the subgroup with CRP measured on POD 2 and POD 4 (*n* = 31; SSI in 4 patients), the mean CRP levels were higher in the SSI group, but the differences were not statistically significant (POD 2: *Z* = 1.227, *p* = 0.223; POD 4: *Z* = 1.492, *p* = 0.139). Similarly, in the subgroup with CRP measured on POD 3 and POD 5 (*n* = 59; SSI in 4 patients), no significant differences were observed (POD 3: *Z* = −0.397, *p* = 0.702; POD 5: *Z* = −0.501, *p* = 0.629) (Table 4).

**Figure 4:**
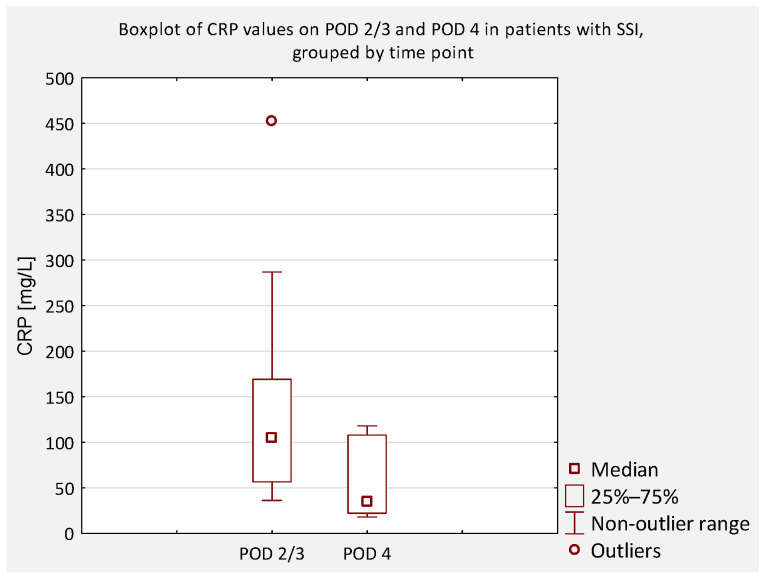
Boxplot of serum CRP concentrations on POD 2/3 and POD 4 in patients with early SSI. No significant difference was observed (*p* = 0.471).

**Figure 5:**
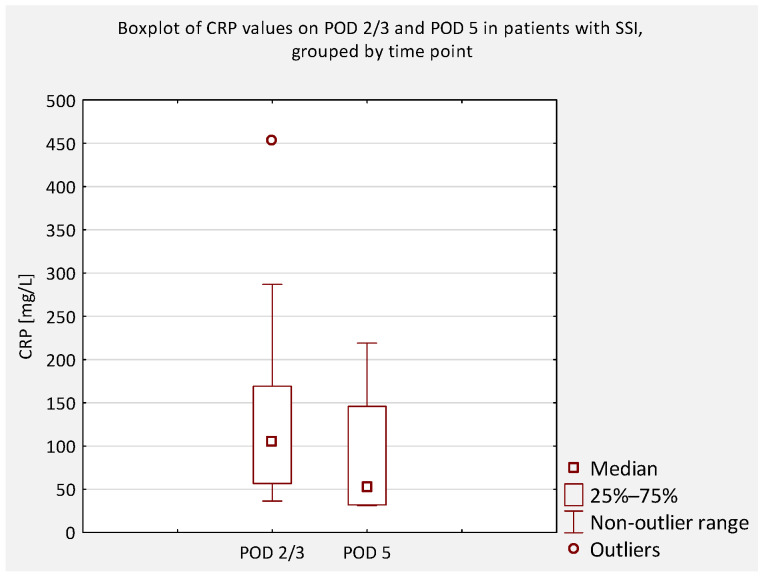
Boxplot of serum CRP concentrations on POD 2/3 and POD 5 in patients with early SSI. No significant difference was observed (*p* = 0.245). A decline in median values was noted, though outliers indicate variability in clinical course.

**Figure 6:**
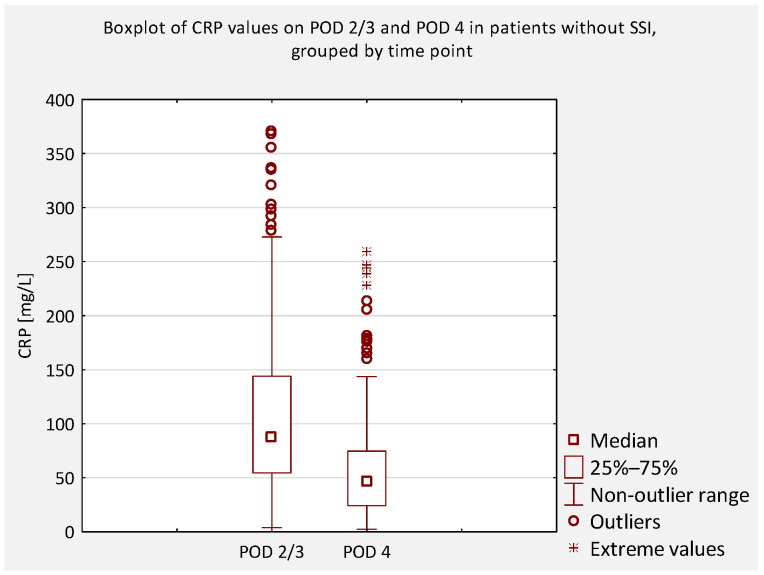
Boxplot of serum CRP concentrations on POD 2/3 and POD 4 in patients without SSI. A significant reduction was observed (*p* < 0.0001), consistent with the expected postoperative decline. Outliers indicate individual variability.

**Figure 7:**
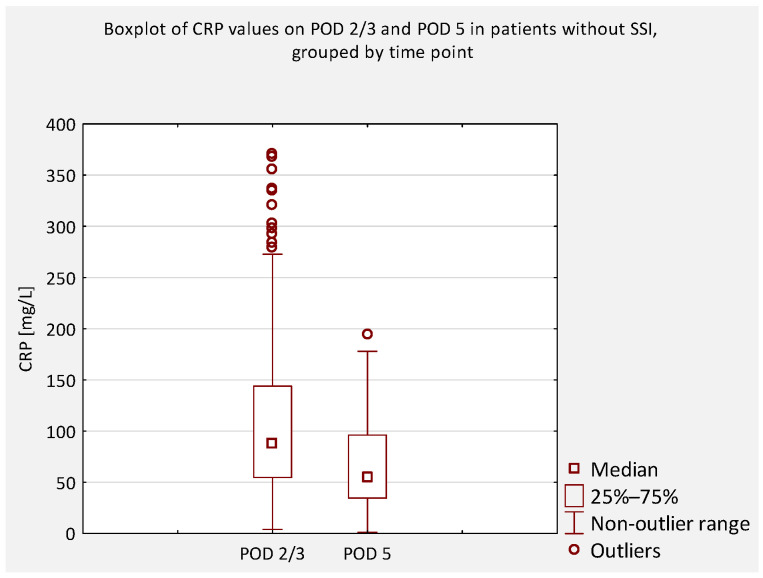
Boxplot of serum CRP concentrations on POD 2/3 and POD 5 in patients without SSI. A significant decrease was observed (*p* < 0.001).

An additional analysis was performed to compare serum CRP concentrations between patients who did not develop an SSI and those who did, regardless of scoliosis type (idiopathic or non-idiopathic). According to previous literature, CRP levels on postoperative days (POD) 2 and 3 are typically similar and correspond to the peak of the postoperative inflammatory response [7]. Therefore, CRP values obtained on POD 2 and 3 were considered equivalent and were jointly compared to values obtained on POD 4 and 5 (Figure 4, Figure 5, Figure 6 and Figure 7).

## 4. Discussion

In this retrospective study, we analyzed postoperative serum CRP concentrations in patients undergoing surgical correction of idiopathic (IS) and non-idiopathic scoliosis (N-IS) to evaluate its utility as an early marker of surgical site infection (SSI). The overall incidence of early SSI was 3.91%, with a higher rate observed in the N-IS group (8.89%) compared with the IS group (2.24%).

A statistically significant decline in CRP levels was observed in patients without SSI, whereas no consistent trend was found in those with confirmed infections. Although most patients showed a typical postoperative CRP decrease, considerable individual variability was noted, even in the absence of complications. These findings underscore the importance of clinical context when interpreting biochemical markers.

Our results align with previous studies showing that serum CRP typically peaks within the first 2–3 postoperative days and gradually declines thereafter if no complications occur [12,13]. This characteristic pattern was also observed in the majority of our non-SSI patients, confirming a normal postoperative inflammatory response. In most patients who subsequently developed SSI, no secondary increase in CRP was observed on postoperative days (POD) 4 or 5; instead, CRP levels declined despite the later occurrence of infection.

However, as reported by others, the use of CRP alone as a diagnostic tool for SSI remains limited in both adult and pediatric spinal surgery populations [6,14,15,16,17,18]. In our cohort of scoliosis patients who subsequently developed SSI, the CRP trajectory between POD 2/3 and POD 4/5 was not indicative of infection. In 11 of 13 evaluable cases, CRP levels declined despite later SSI, whereas only two showed a rise. Thus, early postoperative CRP dynamics do not reliably discriminate between patients who will and will not develop SSI. A secondary rise in CRP after an initial decline has been proposed as a potential indicator of infection [8,11]. Monitoring CRP kinetics—rather than relying on absolute values—may help in the early identification of SSI. A failure of CRP to decrease or a renewed increase should prompt clinical reassessment.

Several studies have investigated alternative biomarkers. Syvänen et al. demonstrated that procalcitonin (PCT) outperformed CRP in differentiating infection from the systemic inflammatory response in adolescents undergoing scoliosis surgery [19]. Aljabi et al. similarly found PCT to be superior to other inflammatory markers in detecting SSI following spinal surgery [20]. Miyamoto et al. showed that lower albumin levels on POD 1 were associated with increased infection risk [6], while Qureshi et al. and Badin et al. emphasized the role of preoperative nutritional status, particularly hypoalbuminemia, in SSI risk after spinal surgery [21,22].

This study has several limitations. First, the number of SSI cases was small, limiting statistical power. Second, changes in the CRP measurement protocol over time may have introduced heterogeneity, although we addressed this analytically. Third, the retrospective design limits control over confounding. Most importantly, CRP was measured only twice in the early postoperative period (POD 2/3 and 4/5), as dictated by our hospital protocol, which was informed by the published literature. This approach may have prevented assessment of the full CRP kinetics or detection of a secondary rise potentially linked to early SSI. Recognition of this limitation in routine care was a key motivation for conducting the present study.

The post hoc power analysis indicated that detecting small differences in CRP dynamics would require a larger sample size. This consideration should be taken into account in future studies aiming to refine the diagnostic role of CRP in early SSI detection. While the present study may have been underpowered to detect subtle effects, the overall findings provide valuable information regarding the limited diagnostic utility of CRP in this context. Despite these limitations, the study has notable strengths: all surgeries were performed in a single center by the same experienced surgeon, with standardized operative technique and perioperative antibiotic prophylaxis.

Future prospective studies with larger SSI cohorts are needed to validate the predictive value of CRP in scoliosis surgery. Integrating CRP kinetics with other biomarkers (e.g., PCT) or imaging modalities may enhance early SSI detection.

## 5. Conclusions

Although CRP is a commonly used marker of postoperative inflammation, this study shows that it is not reliable as a standalone tool for early detection of surgical site infection (SSI) following scoliosis correction. While a significant decline in CRP levels was observed in patients without SSI, the variability of values in both infected and non-infected patients limits its diagnostic utility. Clinical assessment remains essential for early SSI identification. The use of a standardized surgical technique, a single-surgeon approach, and uniform antibiotic prophylaxis strengthens the validity of these findings.

## Figures and Tables

**Table 1:** Demographic characteristics of patients with idiopathic scoliosis (IS) and non-idiopathic scoliosis (N-IS).

Parameter	IS (*n* = 268)	N-IS (*n* = 90)	*p* Value
Age (years) Mean (range)	15.78	15.01	0.131
	(7.59–36.99)	(7.18–46.88)	
BMI (kg/m^2^) Mean (range)	19.81 (11.92–35.63)	17.72 (9.28–37.28)	<0.01
Sex (M/F)	65/203	28/62	0.252
Weight (kg) Mean (range)	65/203	28/62	0.252
	53.73 (19–115)	41.5 (13.2–92)	0.077

**Table 2:** Postoperative serum CRP concentrations on POD 2 and POD 4 in idiopathic scoliosis (IS) and non-idiopathic scoliosis (N-IS), with and without early surgical site infection (SSI); data are mean ± SD [min–max], mg/L. SSI (+) indicates presence of early SSI; SSI (−) indicates absence of early SSI.

Cohort	SSI Status	*n* (POD2)	POD2 (mgl/L)	*n* (POD4)	POD4 (mg/L)	*p* Value (No SSI vs. SSI)
IS	SSI (−)	208	78.93 ± 52.40 (5.9–320.5)	208	51.72 ± 42.41 (2.6–258.8)	<0.01
IS	SSI (+)	6	61.96 ± 27.10 (36.3–105.3)	6	55.93 ± 44.88 (18.1–118.1)	0.89
N-IS	SSI (−)	54	139.28 ± 79.26 (3.8–370.4)	55	85.15 ± 60.08 (2.4–246.2)	<0.01
N-IS	SSI (+)	4	191.88 ± 180.20 (56.7–452.7)	4	88.90 ± 88.82 (32.2–219)	0.069

**Table 3:** Postoperative serum CRP concentrations on POD 3 and POD 5 in idiopathic scoliosis (IS) and non-idiopathic scoliosis (N-IS), with and without early surgical site infection (SSI); data are mean ± SD [min–max], mg/L.

Cohort	SSI Status	*n* (POD3)	POD3 (mg/L)	*n* (POD5)	POD5 (mg/L)	*p* Value (No SSI vs. SSI)
IS	No SSI	53	148.44 ± 73.52	53	59.97 ± 40.76	<0.01
IS	With SSI	-	-	-	-	-
N-IS	No SSI	26	177.68 ± 79.88	27	81.58 ± 44.61	<0.01
N-IS	With SSI	4	197.4 ± 89.69	4	124.83 ± 51.12	0.069

**Table 4:** Between-group comparisons of serum CRP (No SSI vs. SSI) by cohort and postoperative day (POD); Mann–Whitney U test (*Z*, *p*).

Cohort	Measurement Set	POD	*n* (No SSI)	*n* (SSI)	*Z* Score	*p* Value
IS	POD 2 & 4	POD 2	208	6	0.601	0.556
IS	POD 2 & 4	POD 4	208	6	−0.094	0.924
N-IS	POD 2 & 4	POD 2	27	4	1.227	0.223
N-IS	POD 2 & 4	POD 4	27	4	1.492	0.139
N-IS	POD 3 & 5	POD 3	55	4	−0.397	0.702
N-IS	POD 3 & 5	POD 5	55	4	−0.501	0.629

## Data Availability

The data presented in this study are available on request from the corresponding author. The data are not publicly available due to privacy and ethical restrictions.

## Abbreviations

The following abbreviations are used in this manuscript:

SSI	Surgical site infection
CRP	C-Reactive protein
POD	Postoperative day
BMI	Body mass index
PCT	Procalcitonin
IS	Idiopathic scoliosis
N-IS	Non-idiopathic scoliosis
SD	Standard deviation

## References

[1] Mackenzie W.G., Matsumoto H., Williams B.A., Corona J., Lee C., Cody S.R., Covington L., Saiman L., Flynn J.M., Skaggs D.L. (2013). Surgical site infection following spinal instrumentation for scoliosis: A multicenter analysis of rates, risk factors, and pathogens. J. Bone Jt. Surg..

[2] Li Y., Glotzbecker M., Hedequist D. (2012). Surgical site infection after pediatric spinal deformity surgery. Curr. Rev. Musculoskelet. Med..

[3] Matsumoto H., Larson E.L., Warren S.I., Hammoor B.T., Bonsignore-Opp L., Troy M.J., Barrett K.K., Striano B.M., Li G., Terry M.B. (2022). A clinical risk model for surgical site infection following pediatric spine deformity surgery. J. Bone Jt. Surg..

[4] Ho C., Skaggs D.L., Weiss J.M., Tolo V.T. (2007). Management of infection after instrumented posterior spine fusion in pediatric scoliosis. Spine.

[5] Berríos-Torres S.I., Umscheid C.A., Bratzler D.W., Leas B., Stone E.C., Kelz R.R., Reinke C.E., Morgan S., Solomkin J.S., Mazuski J.E. (2017). Centers for Disease Control and Prevention guideline for the prevention of surgical site infection, 2017. JAMA Surg..

[6] Miyamoto A., Tanaka M., Paz Flores A.O., Yu D., Jain M., Heng C., Komatsubara T., Arataki S., Oda Y., Shinohara K. (2024). Predicting surgical site infections in spine surgery: Association of postoperative lymphocyte reduction. Diagnostics.

[7] Kang B.U., Lee S.H., Ahn Y., Choi W.C., Choi Y.G. (2010). Surgical site infection in spinal surgery: Detection and management based on serial C-reactive protein measurements. J. Neurosurg. Spine.

[8] Hoeller S., Roch P.J., Weiser L., Hubert J., Lehmann W., Saul D. (2021). C-reactive protein in spinal surgery: More predictive than prehistoric. Eur. Spine J..

[9] White J., Kelly M., Dunsmuir R. (1998). C-reactive protein level after total hip and total knee replacement. J. Bone Jt. Surg. Br..

[10] Schwarz E.M., Parvizi J., Gehrke T., Aiyer A., Battenberg A., Brown S.A., Callaghan J.J., Citak M., Egol K., Garrigues G.E. (2019). 2018 International Consensus Meeting on Musculoskeletal Infection: Research priorities from the general assembly questions. J. Orthop. Res..

[11] Fujita R., Takahata M., Kokabu T., Oda I., Kajino T., Hisada Y., Takeuchi H., Iwasaki N. (2019). Retrospective study to evaluate the clinical significance of a second rise in C-reactive protein level following instrumented spinal fusion surgery. J. Orthop. Sci..

[12] Rohe S., Böhle S., Matziolis G., Jacob B., Wassilew G., Brodt S. (2023). C-reactive protein during the first 6 postoperative days after total hip arthroplasty cannot predict early periprosthetic infection. Arch. Orthop. Trauma Surg..

[13] Pinchuk A., Luchtmann M., Neyazi B., Dumitru C.A., Stein K.P., Sandalcioglu I.E., Rashidi A. (2024). Is an elevated preoperative CRP level a predictive factor for wound healing disorders following lumbar spine surgery?. J. Pers. Med..

[14] Dowdell J., Brochin R., Kim J., Overley S., Oren J., Freedman B., Cho S. (2018). Postoperative spine infection: Diagnosis and management. Glob. Spine J..

[15] Konishi K., Sano H., Kawano Y., Moroi T., Takeuchi T., Takahashi M., Hosogane N. (2024). Factors related to surgical site infection in spinal instrumentation surgery: A retrospective study in Japan. Asian Spine J..

[16] Gelderman S.J., Faber C., Kampinga G.A., Jutte P.C., Ploegmakers J.J.W., Glaudemans A.W.J.M., Wouthuyzen-Bakker M. (2023). A high prevalence of Cutibacterium acnes infections in scoliosis revision surgery: A diagnostic and therapeutic dilemma. Spine Deform..

[17] Imabayashi H., Miyake A., Chiba K. (2022). Establishment of a suitable combination of serological markers to diagnose surgical site infection following spine surgery: A novel surgical site infection scoring system. J. Orthop. Sci..

[18] Hu X., Lieberman I.H. (2018). Revision spine surgery in patients without clinical signs of infection: How often are there occult infections in removed hardware?. Eur. Spine J..

[19] Syvänen J., Peltola V., Pajulo O., Ruuskanen O., Mertsola J., Helenius I. (2014). Normal behavior of plasma procalcitonin in adolescents undergoing surgery for scoliosis. Scand. J. Surg..

[20] Aljabi Y., Manca A., Ryan J., Elshawarby A. (2019). Value of procalcitonin as a marker of surgical site infection following spinal surgery. Surgeon.

[21] Qureshi R., Rasool M., Puvanesarajah V., Hassanzadeh H. (2018). Perioperative nutritional optimization in spine surgery. Clin. Spine Surg..

[22] Badin D., Leland C.R., Matsumoto H., Roye B., Vitale M., Flynn J., Samdani A., Larson A.N., Yaszay B., Pahys J. (2022). Best practice guidelines for surgical site infection in high-risk pediatric spine surgery: Definition, prevention, diagnosis, and treatment. J. Pediatr. Orthop..

